# Protective Effect on Pancreatic Acinar Cell by Maintaining Cardiac Output in Canine Heart Failure Model With Decreased Pancreatic Blood Flow

**DOI:** 10.3389/fvets.2022.925847

**Published:** 2022-07-15

**Authors:** Aritada Yoshimura, Takahiro Ohmori, Daiki Hirao, Miori Kishimoto, Tomoko Iwanaga, Naoki Miura, Kazuhiko Suzuki, Ryuji Fukushima

**Affiliations:** ^1^Animal Medical Center, Faculty of Agriculture, Tokyo University of Agriculture and Technology, Fuchu, Japan; ^2^Cooperative Department of Veterinary Medicine, Tokyo University of Agriculture and Technology, Fuchu, Japan; ^3^Veterinary Teaching Hospital, Joint Faculty of Veterinary Medicine, Kagoshima University, Kagoshima, Japan

**Keywords:** cardiac output, dog, heart failure, hypoperfusion, ischemia, pancreas, pancreatic acinar cell, pimobendan

## Abstract

Heart failure cause hypoperfusion-induced damage to abdominal organs due to decreased cardiac output (CO). Using a model dog with heart failure caused by rapid ventricular pacing (RVP), we have previously demonstrated that a decrease in CO reduces pancreatic blood flow (PBF). Furthermore, we have revealed that pancreatic acinar cell atrophy, which is a change in the pre-stage of pancreatitis was caused. However, the mechanism by which pancreatic acinar cell atrophy was caused in RVP dogs remains unknown. This study aimed to clarify the association between cardiac function, PBF, and histopathological changes in pancreatic acinar cells by administrating pimobendan, which increase CO, to RVP dogs. RVP dogs were divided into the control group (no medication, *n* = 5) and the pimobendan group (pimobendan at 0.25 mg/kg BID, *n* = 5). Non-invasive blood pressure measurement, echocardiography, and contrast-enhanced ultrasonography for PBF measurement were performed before initiating RVP and at 4 weeks after initiating RVP (4 weeks). At 4 weeks, the decreases in CO, mean blood pressure and PBF due to RVP were suppressed in pimobendan group. Furthermore, histopathological examination showed no changes in pancreatic acinar cells in the pimobendan group. Overall, it was clarified that the decrease in PBF due to cardiac dysfunction was a direct cause of pancreatic acinar cell atrophy. This suggests that maintaining PBF is clinically important for treating dogs with heart failure. In addition, these findings offer a reliable basis for developing new therapeutic strategies for heart failure in dogs, that is, pancreatic protection.

## Introduction

Heart diseases, such as cardiomyopathy and valvular disease, reduce cardiac function and make it challenging to pump sufficient arterial blood, eventually leading to heart failure. Heart failure is defined as a condition in which various clinical signs appear due to decreased cardiac output (CO) (1). In particular, severely decreased CO can cause various ischemic/hypoperfusion injuries or disorders in the abdominal organs (2–4). Among the abdominal organs, the pancreas is known to be vulnerable to hypoperfusion (5). Previously, we developed a heart failure model in a dog by performing rapid ventricular pacing (RVP) and demonstrated the association between cardiac function and pancreatic perfusion by evaluating the change in pancreatic blood flow (PBF) in these animals (6). The findings of the study clarified that a decrease in CO lowers blood pressure and reduces PBF (6). Furthermore, examination of the pancreatic tissue revealed that pancreatic acinar cell atrophy, which is a change in the pre-stage of pancreatitis, was caused (7). However, the mechanism by which RVP causes pancreatic acinar cell atrophy in dogs remains unknown. Furthermore, the causal association between the decrease in PBF and this histopathological change has not been clarified. We hypothesized that if we could mitigate the decrease in PBF following the RVP-induced decrease in CO, then the association between the decrease in PBF and pancreatic acinar cell atrophy could be clarified by histopathological examination of the pancreas of the test animal.

One way to increase CO is to increase cardiac contractility (8). Conventional cardiotonic agents such as cardiac glycosides and catecholamines exert pharmacological effects by increasing the Ca^2+^ concentration in cardiomyocytes (8–10). However, these drugs both have arrhythmogenic effects due to intracellular Ca^2+^ overload and risk damaging the cardiomyocytes (8). Therefore, the emergence of new alternative drugs to treat heart failure is expected.

Recently, pimobendan, a calcium sensitizer, was widely used as a cardiotonic drug to treat heart failure in dogs (11–15). Pimobendan exerts a positive inotropic effect by enhancing the sensitivity of myocardial troponin C to Ca^2+^ (8, 16). This makes it possible to increase CO without increasing the heart rate (HR) (17, 18). Additionally, pimobendan relaxes vascular smooth muscles by inhibiting phosphodiesterase III; the blood pressure is thus maintained by increasing CO (18). Previously, pimobendan also improved renal blood flow in dogs with experimentally induced myxomatous mitral valve degeneration (19). Therefore, we suspected that administering pimobendan could mitigate the decrease in PBF in RVP dogs.

Pimobendan was administered to RVP dogs in this study with the expectation that CO would be increased, despite the actions of RVP. Subsequently, we examined whether the decrease in PBF is mitigated and examined the changes observed in the morphology of pancreatic acinar cells at that time. This study aimed to clarify the association between cardiac function, PBF, and histopathological changes in pancreatic acinar cells.

## Materials and Methods

Healthy beagle dogs (five male and five female) were randomly divided into two groups. Under general anesthesia, epicardial pacemaker leads were placed in each animal. These dogs were 2–3 years old and weighed between 9.3 and 11.2 kg (median: 9.7 kg). Pimobendan (Pimobeheart®, Kyoritsu Seiyaku Corporation, Tokyo, Japan) was administered to one group at a dose of 0.25 mg/kg BID. This dose is generally used to improve clinical signs associated with chronic heart failure due to mitral valve degeneration. The drug was orally administered at 8 a.m. and 4 p.m. The group without medication was defined as the control group, and the group that received pimobendan was defined as the pimobendan group. RVP was implemented in both groups for a period of 4 weeks.

The procedure of pacemaker implantation and pancreatic tissue sampling, in addition to evaluation of cardiac function, PBF, and pancreatic tissue, were the same as described in our previous reports (6, 7). Therefore, the methods for evaluating the cardiac function, PBF, and pancreatic tissue will only be briefly described here. These evaluations were conducted before initiating RVP (baseline) and at 4 weeks after initiating RVP (4 weeks). Cardiac function was evaluated using echocardiography and non-invasive blood pressure measurements using the same device (Vivid E 95 and 7s probe, GE Healthcare Japan Co., Tokyo, Japan, and BP-100D, Fukuda M-E Kogyo Co., Ltd., Tokyo, Japan.). Moreover, PBF was evaluated using contrast-enhanced ultrasonography (CEUS). The items measured by echocardiography and non-invasive blood pressure measurements were as follows. Left atrial to aortic ratio (LA/Ao) was determined from B mode measurements. Left ventricular end-diastolic dimension (LVIDd), left ventricular end-systolic dimension (LVIDs), and fractional shortening (FS) was measured using the M mode. Additionally, ratio of pre-ejection period to ejection time (PEP/ET), stroke volume (SV), CO, and cardiac index (CI) were measured using the Doppler mode. Systolic blood pressure (SBP), mean blood pressure (MBP), and diastolic blood pressure (DBP) were measured by oscillometric methods. Systemic vascular resistance (SVR) was calculated using CO, MBP, and assumed central venous pressure (CVP). CVP was hypothesized to be 5 mmHg at baseline, and 5, 10, or 15 mmHg at 4 weeks, based on previous reports in the same heart failure model (20).

**Figure 1:**
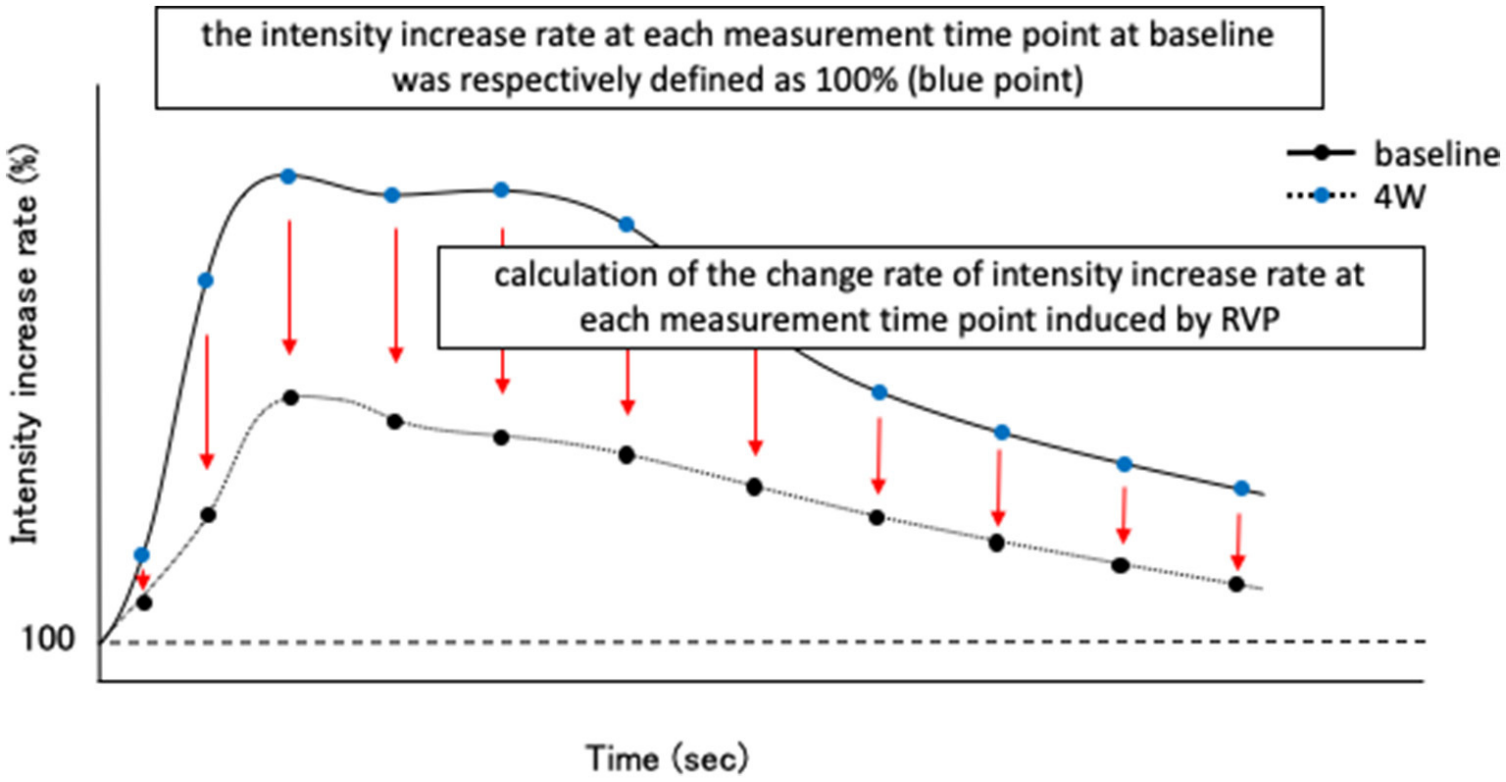
Schematic diagram of evaluation method of intensity reduction level due to rapid ventricular pacing (RVP) in contrast-enhanced ultrasonography (CEUS). First, before initiating RVP (baseline) in both control group and pimobendan group, the intensity increase rate at each measurement time point after Sonazoid® administration (in this study, from 0 to 300 s) was respectively defined as 100% (blue point). Then, the change rate of intensity increase rate at each time point at 4 weeks after initiating RVP (4 weeks) was calculated.

Contrast-enhanced ultrasonography was performed using the same device (Logiq7 and 9L probe, GE Healthcare Japan, Tokyo) and the same contrast agents (perflubutane microbubbles; Sonazoid®, Daiichi-Sankyo Co., Ltd., Tokyo, Japan) as in the previous study. Blood flow delivers the contrast media and the rate of inflow of the bubbles increases the intensity of the CEUS signal. We measured the signal intensity at 5-s intervals from 0 to 180 s and at 10-s intervals from 180 to 300 s after administration of the contrast agents. Similar to our previous report, we defined the intensity before contrast administration as 100% and created a time-intensity increase rate curve (TIC) from 0 to 300 s. Moreover, time to peak (TP), time to initial up-slope (TTU), time to washout (TTW), peak intensity (PI), and area under the curve (AUC) were calculated as blood flow index parameters. Then, the degree of decrease in the intensity due to RVP was examined. Specifically, at baseline in both groups, the intensity increase rate at each measurement time point from 0 to 300 s was respectively defined as 100% (Figure 1). Moreover, we calculated the change rate of intensity increase rate at each measurement time point induced by RVP for 4 weeks (Figure 1).

Pancreatic tissue was evaluated by histopathological examination using hematoxylin and eosin staining. A semiquantitative evaluation was performed based on the extent of zymogen granules in pancreatic acinar cells of 20 consecutive microscopic fields (×400). The following scoring was applied: “0” for <5% pancreatic acinar cells containing zymogen granules, “1” for 5–25%, “2” for 26–50%, and “3” for 51% or more. Moreover, immunohistochemical staining was performed targeting trypsinogen in pancreatic acinar cells using the same anti-trypsin antibodies (×50, rabbit monoclonal, Abcam Plc, Cambridge, UK) as in previous report.

The RVP for inducing heart failure was set at 4 V and 260 beats/min for 4 weeks. This prospective study was performed with approval from Tokyo University of Agriculture and Technology Animal Experiment Committee (Approval number: 31-2).

### Statistical Analysis

Statistical analysis was conducted using BellCurve for Excel (Social Survey Research Information Co., Ltd., Tokyo, Japan). The normality of data was tested with the Shapiro–Wilk test. The change rate of intensity increase rate at each time point for CEUS and histopathological scoring data are shown as the mean ± standard error. Other data are shown as mean ± standard deviation. Intra-group comparisons in echocardiographic measurements, blood pressure value, CEUS measurements other than the change in the signal intensity, and histopathological scores were performed using paired *t*-test or Wilcoxon's signed-rank test. Moreover, inter-group comparisons in these data were performed using the unpaired *t*-test or Mann–Whitney *U*-test. Regarding the change rate of intensity increase rate at each time point, intra-group comparisons were conducted using the Friedman test with *post-hoc* Mann–Whitney *U*-test. For all tests, a *P* value <0.05 was considered statistically significant.

## Results

### Echocardiography

The results of echocardiographic measurements are shown in Table 1. FS decreased significantly at 4 weeks compared to baseline readings in both groups (*P* < 0.01 in both groups). However, at 4 weeks, FS in the pimobendan group was significantly greater than in the control group (*P* < 0.01). In the pimobendan group, compared to baseline, no significant difference was shown in SV, CO, and CI at 4 weeks. In contrast, in the control group, SV, CO, and CI were significantly smaller at 4 weeks than at baseline (SV; *P* < 0.01, CO and CI; *P* < 0.05). Moreover, at 4 weeks, CO and CI in the pimobendan group were significantly greater compared to the control group (*P* < 0.05).

**Table 1:** Values of echocardiography and blood pressure measurement.

	**Group**	**Baseline**	**4 weeks**
HR (bpm)	Control	101 ± 10	150 ± 8^*^
	Pimobendan	108 ± 15	137 ± 25
LA/Ao	Control	1.4 ± 0.1	1.9 ± 0.2^**^
	Pimobendan	1.3 ± 0.1	1.7 ± 0.1^**^
LVIDd (mm)	Control	30.2 ± 2.4	39.9 ± 3.6^**^
	Pimobendan	32.4 ± 1.5	40.4 ± 2.4^**^
LVIDs (mm)	Control	17.8 ± 1.9	35.7 ± 2.9^**^
	Pimobendan	20.2 ± 2.2	32.8 ± 2.1^**^
FS (%)	Control	40.9 ± 4.6	10.6 ± 2.5^**^
	Pimobendan	37.6 ± 5.8	18.8 ± 3.7^**^^,^^##^
PEP/ET	Control	0.3 ± 0.0	0.5 ± 0.0^**^
	Pimobendan	0.3 ± 0.0	0.4 ± 0.1^**^^,^^#^
SV (ml)	Control	18.3 ± 3.3	10.3 ± 1.5^**^
	Pimobendan	19.4 ± 3.0	16.4 ± 5.9
CO (L/min)	Control	2.1 ± 0.2	1.5 ± 0.4*
	Pimobendan	2.1 ± 0.3	2.1 ± 0.5^#^
CI (L/min/m^2^)	Control	4.3 ± 0.6	3.0 ± 0.9*
	Pimobendan	4.4 ± 0.7	4.5 ± 1.0^#^
SBP (mmHg)	Control	142.7 ± 12.7	102.6 ± 7.8^**^
	Pimobendan	147.7 ± 17.2	124.0 ± 10.2^##^
MBP (mmHg)	Control	97.9 ± 4.1	72.9 ± 12.0*
	Pimobendan	110.0 ± 12.3	88.1 ± 7.9^#^
DBP (mmHg)	Control	77.2 ± 6.5	57.9 ± 13.5
	Pimobendan	84.7 ± 10.0	71.6 ± 9.4

*HR, heart rate; LA/Ao, left atrial to aortic ratio; LVIDd, left ventricular end-diastolic dimension; LVIDs, left ventricular end-systolic dimension; FS, fractional shortening; PEP/ET, ratio of pre-ejection period to ejection time; SV, stroke volume; CO, cardiac output; CI, cardiac index; SBP, systolic blood pressure; MBP, mean blood pressure; DBP, diastolic blood pressure; baseline, before initiating rapid ventricular pacing; 4 weeks, 4 weeks after initiating rapid ventricular pacing*.

* *, P < 0.05 vs. baseline*;

** *, P < 0.01 vs. baseline*;

# *, P < 0.05 vs. control*;

## *, P < 0.01 vs. control. All values are expressed as a mean ± standard deviation*.

Left atrial to aortic ratio, LVIDd, LVIDs, and PEP/ET were significantly greater at 4 weeks than at baseline in both groups (*P* < 0.01 in both groups). However, PEP/ET at 4 weeks in the pimobendan group was significantly smaller compared to the control group (*P* < 0.05). In the pimobendan group, no significant difference was shown in HR between baseline and 4 weeks. In contrast, HR was significantly greater at 4 weeks than at baseline in the control group (*P* < 0.05).

### Blood Pressure Measurement

In the pimobendan group, no significant difference was shown in SBP and MBP between baseline and 4 weeks. On the other hand, these pressures were significantly lower at 4 weeks than at baseline in the control group (SBP, *P* < 0.01; MBP, *P* < 0.05). Furthermore, SBP and MBP at 4 weeks in the pimobendan group were significantly greater compared to the control group (SBP, *P* < 0.01; MBP, *P* < 0.05). There was no significant difference in DBP between the groups or within each group (Table 1). Assuming that CVP at baseline is 5 mmHg, and at 4 weeks is 5, 10, or 15 mmHg, no significant difference was shown in SVR between baseline and 4 weeks in the pimobendan group. However, when CVP at 4 weeks was assumed to be 10 or 15 mmHg, the value tended to decrease (10 mmHg; *P* = 0.097, 15 mmHg; *P* = 0.058). Moreover, no significant difference was shown in SVR between baseline and 4 weeks in the control group, at any combination of CVP values (Table 2).

**Table 2:** Systemic vascular resistance when central venous pressure is postulated to be 5–15 mmHg.

	**mmHg**	**Group**	**Period**	
			**Baseline**	**4 weeks**
	5	Control	3,501 ± 401	3,554 ± 887
		Pimobendan	4,149 ± 917	3,232 ± 778
SVR (dynes × s × cm^−5^)	10	Control		3,293 ± 849
		Pimobendan		3,037 ± 732
	15	Control		3,032 ± 811
		Pimobendan		2,841 ± 688

*SVR, systemic vascular resistance; baseline, before initiating rapid ventricular pacing; 4 weeks, 4 weeks after initiating rapid ventricular pacing. All values are expressed as a mean ± standard deviation*.

### Contrast-Enhanced Ultrasonography

**Figure 2:**
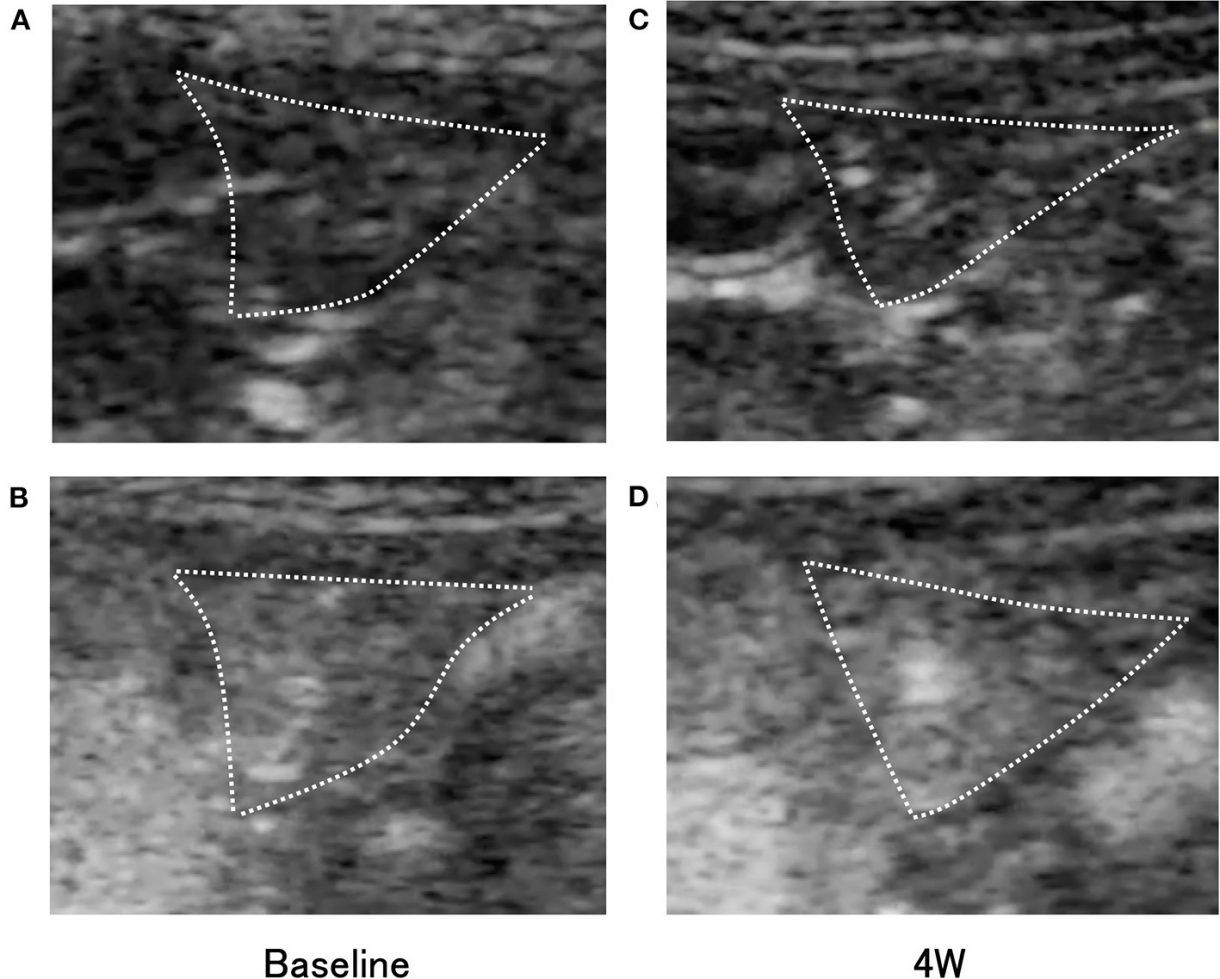
Transverse contrast-enhanced ultrasonography (CEUS) images of the right pancreatic lobe (outlined by dotted lines) in the pimobendan group. **(A,B)** show the intensity increase in the pancreas with contrast agent before initiating rapid ventricular pacing (baseline). **(A)** Just after initiation of contrast imaging. **(B)** Peak of intensity increase. **(C,D)** show the intensity increase in the pancreas with contrast agent at 4 weeks after initiating rapid ventricular pacing (4 weeks). **(C)** Just after initiation of contrast imaging. **(D)** Peak of intensity increase.

Subjectively, in the pimobendan group, an increase in pancreatic intensity was observed at 4 weeks and at the baseline (Figure 2). Furthermore, no significant difference was shown in PI and AUC between baseline and 4 weeks. In contrast, in the control group, PI and AUC were significantly smaller at 4 weeks compared with baseline (both *P* < 0.05) (Table 3). As a result, at 4 weeks, AUC and PI in the pimobendan group were significantly greater than in the control group (both *P* < 0.05). In the pimobendan group, no significant difference was shown in TP between baseline and 4 weeks. In contrast, TP was significantly greater at 4 weeks than at baseline in the control group (*P* < 0.05). No significant difference was shown in TTU and TTW in terms of the intra-group and inter-group comparisons. Furthermore, the change rate of intensity increase rate in the pimobendan group was significantly greater than in the control group at almost all time points after Sonazoid® administration (145–160 s and 280 s; *P* < 0.05, other times; *P* < 0.01) (Figure 3).

**Table 3:** Values of parameters of the time-intensity change rate curve in the contrast-enhanced ultrasonography of the pancreas.

	**Group**	**Baseline**	**4 weeks**
PI (%)	Control	111.2 ± 3.4	105.5 ± 1.7^*^
	Pimobendan	109.9 ± 3.9	114.6 ± 7.3^#^
AUC	Control	324.9 ± 126.9	130.3 ± 43.2^*^
	Pimobendan	228.7 ± 90.3	389.7 ± 210.7^#^
TP (s)	Control	55.0 ± 21.2	113.0 ± 34.7^*^
	Pimobendan	79.0 ± 15.9	71.0 ± 10.2^#^
TTU (s)	Control	21.0 ± 4.9	36.0 ± 17.7
	Pimobendan	24.0 ± 3.7	28.0 ± 6.0
TTW (s)	Control	254.0 ± 54.6	211.0 ± 54.1
	Pimobendan	193.0 ± 54.6	214.0 ± 49.6

*PI, peak intensity; AUC, area under the curve; TP, time to peak; TTU, time to initial up-slope; TTW, time to washout; baseline, before initiating rapid ventricular pacing; 4 weeks, 4 weeks after initiating rapid ventricular pacing*.

* *, P < 0.05 vs. baseline*;

# *, P < 0.05 vs. control. All values are expressed as a mean ± standard deviation*.

**Figure 3:**
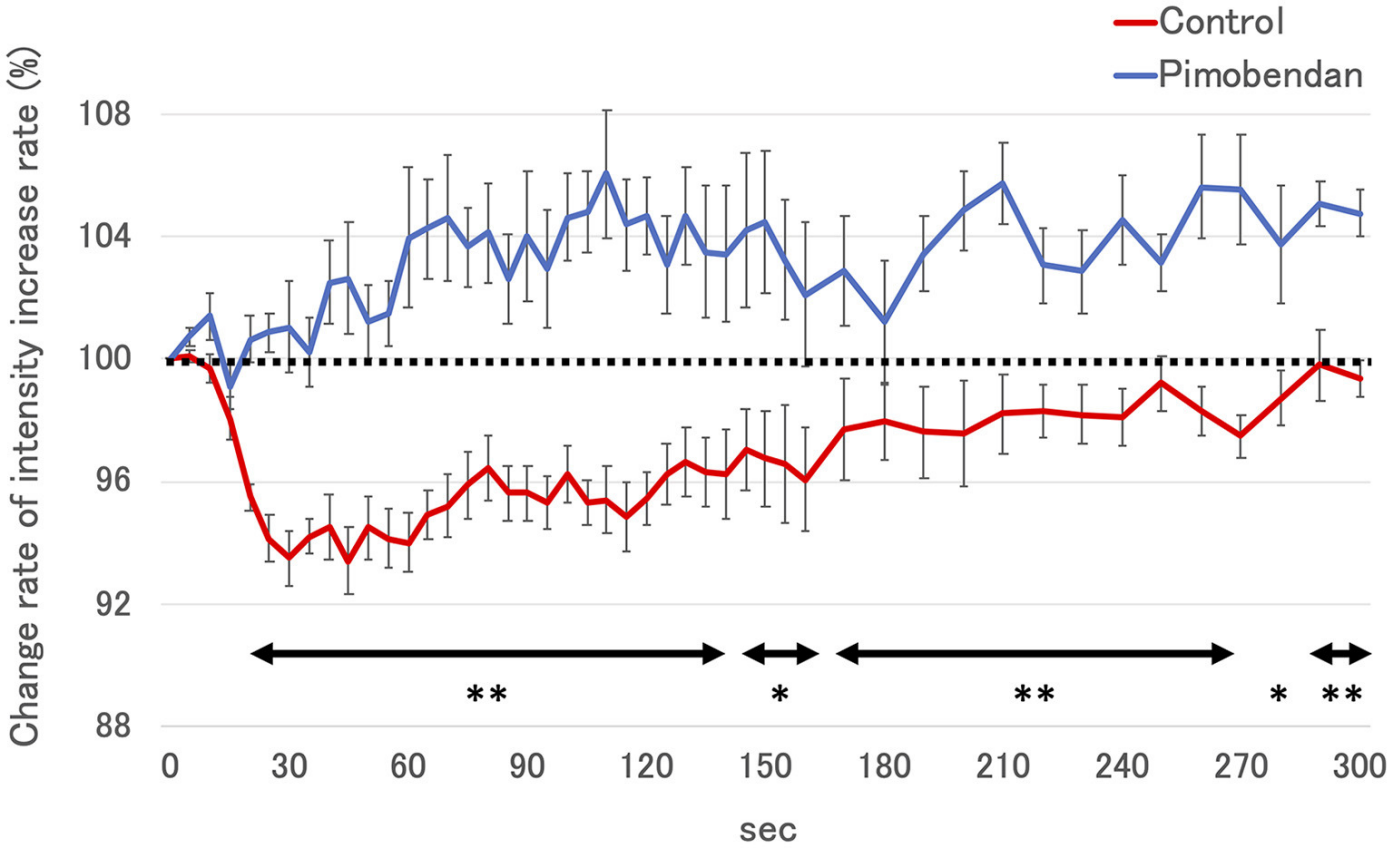
The change rate of intensity increase rate in control group and the pimobendan group in contrast-enhanced ultrasonography (CEUS). The intensity increase rate at each measurement time point after Sonazoid® administration before initiating RVP (baseline) was defined as 100% (dotted line). These values are shown as the mean ± standard error. *; *P* < 0.05 vs. control, **; *P* < 0.01 vs. control.

### Histopathological Examination

**Figure 4:**
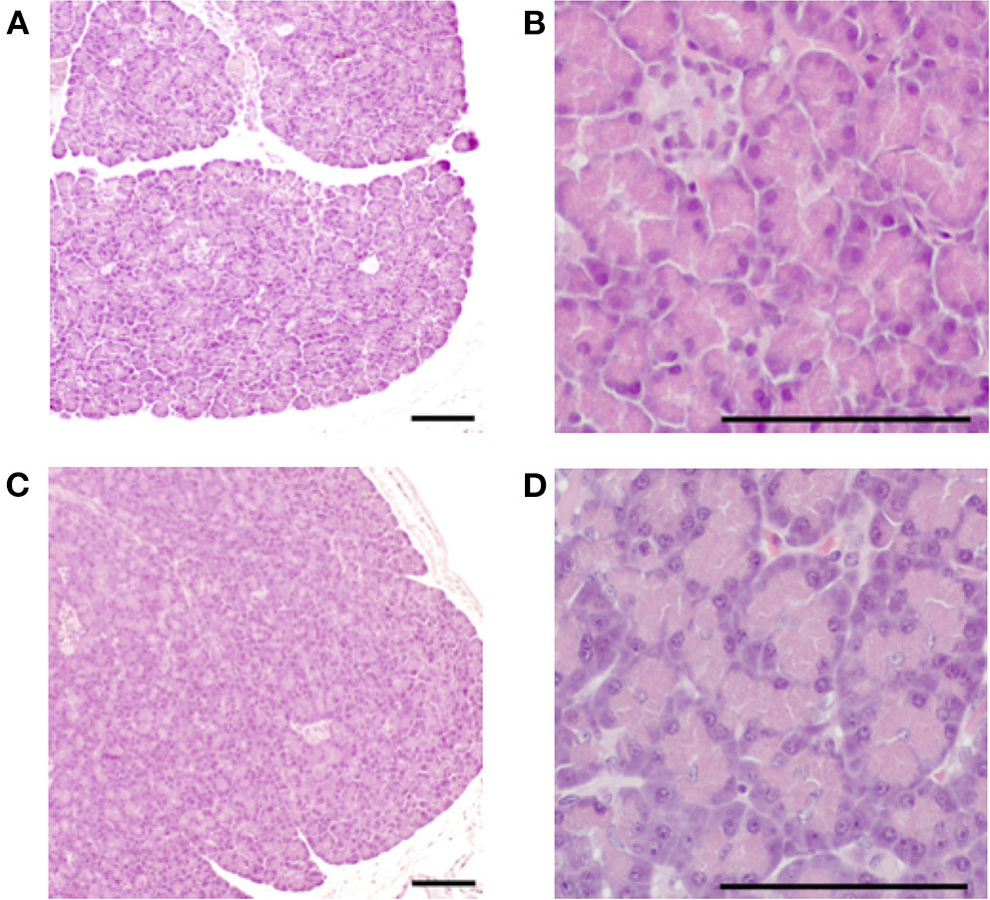
Histopathological image of the pancreas in the pimobendan group. Eosinophilic zymogen granules were observed in the pancreatic acinar cell both before initiating rapid ventricular pacing (baseline) and at 4 weeks after initiating rapid ventricular pacing (4 weeks). **(A,B)** baseline. **(C,D)** 4 weeks. Bars = 100 μm.

No unusual histopathological findings were observed at baseline in either group. In the pimobendan group, no unusual histopathological findings were observed in any animal at 4 weeks (Figure 4). In contrast, pancreatic acinar cell atrophy characterized by degranulation, that is, loss of zymogen granules, was observed in all five dogs at 4 weeks in the control group. Furthermore, this histopathological change was observed in the whole pancreas (*P* < 0.01), and no site-specific difference was shown in the level of the lesion. As a result, the histopathological score at 4 weeks in the pimobendan group was significantly greater compared with that of the control group (*P* < 0.05) (Table 4).

**Table 4:** Histological scoring data of pancreas.

	**Group**	**Baseline**	**4 weeks**
Score	Control	2.7 ± 0.0	1.3 ± 0.1^**^
	Pimobendan	2.7 ± 0.0	2.3 ± 0.0^#^

*Baseline, before initiating rapid ventricular pacing; 4 weeks, 4 weeks after initiating rapid ventricular pacing*.

** *; P < 0.01 vs. baseline*,

# *; P < 0.05 vs. control. All values are expressed as a mean ± standard error*.

### Immunohistological Examination

**Figure 5:**
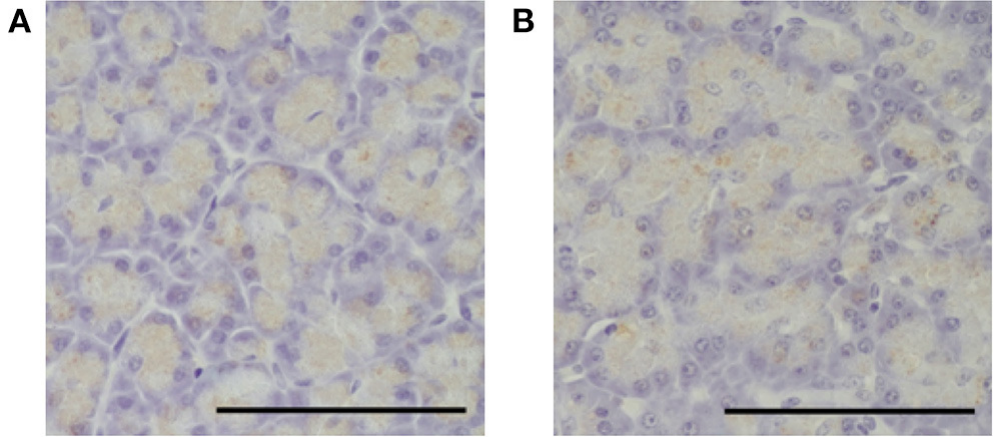
Immunohistochemical image of the pancreas in the pimobendan group. A trypsinogen positive reaction of the zymogen granules was observed in the pancreatic acinar cells both before initiating rapid ventricular pacing (baseline) and at 4 weeks after initiating rapid ventricular pacing (4 weeks). **(A)** baseline. **(B)** 4 weeks. Bars = 100 μm.

A trypsinogen-positive reaction of the zymogen granules was observed in pancreatic acinar cells at baseline in both groups. There was no change in the extent of the trypsinogen-positive reaction at 4 weeks in the pimobendan group (Figure 5). In contrast, at 4 weeks the trypsinogen-positive reaction was attenuated in all animals in the control group.

## Discussion

In this study, similar to our previous reports, we found that a decrease in CO caused a decrease in PBF in unmedicated RVP dogs. Moreover, pancreatic tissue in these dogs showed pancreatic acinar cell atrophy with a decrease in zymogen granules, meaning that the hypoperfusion was significant enough to induce cellular damage. In contrast, in RVP dogs treated with pimobendan, PBF was maintained by maintaining CO. As a result, no histopathological changes were observed in the pancreatic tissue.

Similar to our previous reports, in the unmedicated RVP dogs there was a decrease in FS and an increase in PEP/ET, which indicates a decrease in left ventricular contractility. Moreover, a decrease in CI was observed because of the reduction in left ventricular contractile force. The extent of change in these values was similar to that reported previously (6). Therefore, the RVP dog model used in this study might have good reproducibility of the changed hemodynamics. This heart failure model was determined to be appropriate for investigating the association between cardiac function, PBF, and histopathological changes in the pancreatic acinar cells.

Pimobendan is known to increase CO. Pimobendan has been shown to improve hemodynamics during heart failure due to mitral valve degeneration and dilated cardiomyopathy in dogs, improving quality of life and prolonging the prognosis (11, 12, 14, 15). Moreover, this hemodynamic-improving effect of pimobendan is thought to be mainly due to its positive inotropic effect (18). In this study, the changes in FS and PEP/ET observed in unmedicated RVP dogs were significantly alleviated by pimobendan administration. The decreases in SV, CO, and CI were also suppressed. These results suggest that the positive inotropic effect of pimobendan was effectively exerted on the test animals in this study and could be responsible for the effect of increasing CO. Furthermore, with regard to HR, administration of pimobendan suppressed the increase observed in the control group. Pimobendan is known to increase CO by increasing SV, independent of the increase in HR (17, 18). The results are consistent with this observation and are also considered to have been obtained in this test animal. Furthermore, maintaining CO cannot enhance the compensatory reaction of increasing HR.

Pimobendan relaxes vascular smooth muscle by inhibiting phosphodiesterase III (21). This action causes dilation of the peripheral blood vessels and reduces SVR (21). However, in this study, while the positive inotropic effect was evident the peripheral vasodilatory effect showed a tendency, but no clear effect was observed. Verdouw et al. administered various doses of pimobendan to healthy pigs under general anesthesia and evaluated the positive inotropic and peripheral vasodilatory effects at each dose. In their study, pimobendan first exerted a positive inotropic effect at low doses and a peripheral vasodilatory effect at higher doses (22). Based on these observations, it appears the pimobendan dose used in this study led to a positive inotropic effect on the test animals, but did not reach the point where clear peripheral vasodilation could be observed.

In this study, CEUS was used to measure PBF. CEUS involved injecting microbubbles intravenously as a contrast medium, which acts as a reflection source for ultrasonic waves in capillaries to visualize blood flow and enhance echo intensity in organs (23). By calculating the blood flow index from the TIC, based on this increase in intensity over time, it is possible to quantify the blood flow in the target organ (24, 25). CEUS has proven useful as a non-invasive method to assess PBF (6, 25).

Similar to our previous reports, we observed a significant decrease in PI and AUC in the control group. PI indicates the maximum inflow of microbubbles in the region of interest, while AUC indicates the total inflow of microbubbles into the region within the observation period (24). Therefore, a decrease in these parameters indicates decreased blood flow in the pancreatic parenchyma. Compared to the control group, PI and AUC did not decrease, and PBF was maintained in the RVP dogs treated with pimobendan. This result was further clarified by the fact that a decrease in the intensity in the pimobendan group never occurred at almost all time points after administering Sonazoid®. Furthermore, regarding blood pressure measurements, administering pimobendan prevented the decrease in SBP and MBP. Arterial blood pressure, especially MBP, is thought to reflect the driving pressure that pumps blood to peripheral organs and is a key factor in regulating organ blood flow (26). In this study, we observed no clear change in SVR in the pimobendan group. Therefore, we considered that administering pimobendan maintained PBF because the maintenance of cardiac pump function suppressed the decrease in CO, and as a result alleviated the decrease in MBP.

Histopathological examination showed no changes in pancreatic tissue containing pancreatic acinar cells in the pimobendan group, in which CO and PBF were maintained. In contrast, in the control group, similar to previous reports, pancreatic acinar cell atrophy with a decrease in zymogen granules, and thus the trypsinogen contained therein, was observed over a wide area of the pancreas (7). Therefore, we concluded that a decrease in PBF was a direct cause of pancreatic acinar cell atrophy. Pancreatic acinar cell atrophy due to the decrease in PBF most likely corresponds to the pre-stage histopathological changes caused by chronic pancreatitis (7, 27). Moreover, the histopathological changes in pancreatic tissue may become more severe if the decrease in cardiac function and PBF is prolonged (7). This study showed that administering pimobendan to dogs experiencing decreased cardiac contractile force can prevent the development or aggravation of pancreatic acinar cell injury by helping maintain PBF.

This study has several limitations. First, we measured blood pressure non-invasively. In most cases, compared to invasive measures, this method is inferior in accuracy and immediacy. Therefore, it is possible that dynamic changes in blood pressure and SVR were not completely captured. Second, blood flow in organs other than the pancreas was not measured. In heart failure, biological defense reactions restrict blood flow to the peripheral organs and tissues to maintain blood flow to vital organs such as the brain and heart (28). Moreover, the extent of the decrease in blood flow in these peripheral organs and tissues is not uniform (28). Therefore, by measuring the blood flow of multiple organs simultaneously, it may be possible to better understand the association between changes in cardiac function and PBF. Third, we did not perform a histopathological examination of the heart. In this study, the mechanism by which CO was maintained seems to be due to the positive inotropic effect of pimobendan. However, it has been clarified that pimobendan also increases myocardial perfusion by dilating the coronary arteries (29). Myocardial ischemia due to extreme tachycardia is among the primary factors that cause RVP to decrease myocardial contractile force (30). Therefore, it is possible that the administration of pimobendan suppressed the decrease in cardiac function because the myocardium was protected by maintaining coronary blood flow. It may also be possible to clarify the mechanism of maintained CO by performing a histopathological assessment of the extent of myocardial injury. Finally, this study included only a small number of animals, which might have affected the results.

This study showed that the administration of pimobendan helps maintain PBF by suppressing the decrease in cardiac function in dogs with RVP. Furthermore, pimobendan also suppressed histopathological changes in pancreatic acinar cell atrophy. These results clarified that the decrease in PBF due to cardiac dysfunction was a direct cause of pancreatic acinar cell atrophy. This further suggests that maintaining PBF is clinically important for treating dogs with heart failure. In addition, these findings offer a reliable basis for developing new therapeutic strategies for heart failure in dogs, that is, pancreatic protection.

## Data Availability Statement

The original contributions presented in the study are included in the article/supplementary material, further inquiries can be directed to the corresponding author/s.

## Ethics Statement

The animal study was reviewed and approved by Tokyo University of Agriculture and Technology Animal Experiment Committee (approval number 31-2).

## Author Contributions

AY and RF conceived and designed the experiments. AY, TO, DH, MK, and KS performed the experiments. AY analyzed the data and wrote the manuscript. TI, NM, and RF performed the critical revisions of the manuscript. All authors approved the final manuscript.

## Funding

This work was partially supported by the 2017–2019 the JSPS KAKENHI Grant Number 17K08100 (representative researcher: RF).

## Conflict of Interest

The authors declare that the research was conducted in the absence of any commercial or financial relationships that could be construed as a potential conflict of interest.

## Publisher's Note

All claims expressed in this article are solely those of the authors and do not necessarily represent those of their affiliated organizations, or those of the publisher, the editors and the reviewers. Any product that may be evaluated in this article, or claim that may be made by its manufacturer, is not guaranteed or endorsed by the publisher.

## Abbreviations

AUC: area under the curve
CEUS: contrast-enhanced ultrasonography
CI: cardiac index
CO: cardiac output
CVP: central venous pressure
DBP: diastolic blood pressure
FS: fractional shortening
HR: heart rate
LA/Ao: left atrial to aortic ratio
LVIDd: left ventricular end-diastolic dimension
LVIDs: left ventricular end-systolic dimension
MBP: mean blood pressure
PBF: pancreatic blood flow
PEP/ET: ratio of pre-ejection period to ejection time
RVP: rapid ventricular pacing
SBP: systolic blood pressure
SV: stroke volume
SVR: systemic vascular resistance
TIC: time-intensity increase rate curve
TP: time to peak
TTU: time to initial up-slope
TTW: time to washout.
